# Practical Departments

**Published:** 1905-11-04

**Authors:** 


### PRACTICAL DEPARTMENTS.

Referring to our notice of Messrs. Royles' heating apparatus in our last issue, the address was given as Irlam o' th' Heights, whereas it should have been Irlam, near Manchester.

The huge building containing the new laboratories of the New York Board of Health is now completed. There is accommodation for seventeen investigators and forty-seven assistants.

